# Efficacy of arthroscopic internal fixation with countersunk screw in the treatment of talus fracture

**DOI:** 10.4314/ahs.v23i3.61

**Published:** 2023-09

**Authors:** Bo Sun, Shibo Liu, Xinxin Xue, Yunfeng Gao, Shijie Fu, Pei Wang

**Affiliations:** Deportment of Hand and Foot Surgery, Affiliated hospital of Chengde Medical University, Chengde 067000, Hebei. China

**Keywords:** Arthroscopy, countersunk screw, curative effect, internal fixation, talar fracture

## Abstract

**Objective:**

To explore clinical effects of arthroscopic internal fixation with countersunk screw in the treatment of talus fracture.

**Methods:**

Forty-eight patients with talus fracture treated in hospital of Chengde Medical University from February 2015 to December 2019 were enrolled for present investigation. The patients with talus fracture were randomly assigned into two groups, with twenty-four patients per group. The patients with talus fracture in the observation group were treated with arthroscopic internal fixation with countersunk screw, while the traditional open reduction and internal fixation were applied for the ones in control group. The clinical efficacy of the patients was evaluated three months after the operation, and the preoperative and postoperative ankle joint functions, fracture-healing time, hospital stay, and complications were carefully compared between observation and control group.

**Results:**

A total efficiency as high as 91.67% was showed in observation group, which is distinctly better than the effective rate of control group (66.67%, P<0.05). Before operation, ankle function scores (AOFAS) of control group and observation group is 42.08 ± 4.29 and 41.75±5.31 with no significantly difference (P>0.05); while after the surgery, AOFAS scores of control group is significantly lower than that of observation group: (66.28±7.51 vs. 53.0 ±6.79, P<0.05). Moreover, healing time and hospitalized duration of observation group are 3.19±1.04 months and 3.57±0.97 days, which are also significantly shorter than 4.18±1.25 months and 8.28±2.54 days in control group, respectively, (P < 0.05). And the total complication rate in control group is 20.83%, which is higher than 8.33% in observation group (P >0.05).

**Conclusion:**

Arthroscopic internal fixation with countersunk screw can significantly improve the efficacy and ankle joint functions, shorten the fracture-healing time and hospital stays without increasing the incidence of complications.

## Introduction

Talus is one of the components of the ankle joint and participates in the support of body weight. Talus fracture is one of the serious traumas in foot and ankle surgery, which is mostly caused by traffic accidents and construction injuries. The clinical cases of talus fracture have gradually increased in recent years 1,2,3 Talar fractures can cause deformity if not treated promptly, and even cause ischemic necrosis in severe cases^4^,^5^. The quality of bone, articular cartilage, and soft tissue should be preferentially considered when choosing the treatment. In addition, the surgical plan should be determined according to the range of motion, clinical diagnosis, imaging, and laboratory findings of the joint. With the development of minimally invasive technique, arthroscopic techniques have already been applied in treating different types of ankle fractures. The present investigation deals with exploration of clinical effects of arthroscopic technique on the treatment of talar fractures using countersunk screws for internal fixation, in order to provide novel approaches to treating the talar fractures.

## Materials and methods

### General information

Forty-eight patients with talus fractures, who met the following inclusion and exclusion criteria and were treated at the hospital of Chengde Medical University from February 2015 to December 2019, were enrolled in this study.

**The inclusion criteria as follows:** (1) patient with talus fracture diagnosed by CT and other imaging examinations; (2) patient with conscious mind and normal intelligence; (3) patient with a fresh unilateral closed fracture.

**Exclusion criteria:** (1) patient with abnormal coagulation function; (2) patient with combined other types of ankle injury; (3) patient with combined vascular injury or nerve injury; (4) patient with previous ankle fracture. The patients with talus fracture were randomly assigned into two groups, with twenty-four patients per group. There were eight females and sixteen males in control group; according to causes, there were seven cases of high falling injury, eleven cases of car accident, and six for other reasons; according to Hawkins classification criteria for talar neck fractures: five cases were type I, sixteen cases were type II, and three cases were type III. There were six females and eighteen males in observation group; according to causes, there were seven high falling injuries, eleven car accidents, and six for other reasons; as for Hawkins classification, 5 cases were type I, 16 cases were type II, 3 cases were type III. General information between control and observation group were comparable and there is no significant difference (P > 0.05).

## Methods

Internal fixation and traditional open reduction were applied for the treatment of the patients in control group. All patients were treated with spinal anesthesia in supine position. The surgery was conducted through anteromedial approach, and conventional focus cleaning and reduction at the fracture site were operated through the space between tibialis anterior and tibialis posterior, then the internal fixation screws were turned in from the anterior to the posterior side, and the incision was closed after the fixed position was confirmed by fluoroscopy, thus the surgery was completed. The observation group was treated with arthroscopic internal fixation with countersunk head nail for talus fracture. The anesthesia method was the same as that of the control group; tourniquet was used to compress and stop bleeding at the root of patient's side thigh; the appropriate surgical approach was selected according to the patient's condition, ankle joint was washed under arthroscopy, ion knife was applied for cases such as articular surface wear, exfoliated cartilage, and uneven articular cartilage surface in ankle joint cavity. The bone fragments and cartilage were removed, and the fracture site was reduced under the microscope. Two to three K-wires of 1.5 ~ 2.0 mm a diameter was taken for cross temporary fixation. Then, 2 ~ 4 countersunk screws were taken for firm fixation. The C-arm machine was used to confirm the screw position. After the operation, a drainage tube was placed. After the operation, both groups were fixed with plaster, and the healing condition was re-examined by X-ray once a month. After removing the plaster in 6 ~ 8 weeks, non-weight-bearing rehabilitation training was performed.

### Observation indicators and evaluation methods

(1). Ankle joint function: ankle joint function score (AOFAS) was used before treatment and 3 months after operation^6^. This table mainly assesses the pain, function, and mobility of the patient's ankle joint, and can achieve comprehensive evaluation for the function of the ankle joint. The total table score is 0 to 100, and a higher score was considered as a better function of ankle joint.

(2). The occurrence of complications: record the occurrence of complications such as infection and talar necrosis during treatment.

### Efficacy evaluation criteria

The efficacy of the patients was evaluated three months after operation^7^. Recovered: the patients could walk normally without obvious pain, and there was no impact on normal life or work; markedly effective: basically, the patients walked normally with a slight pain, which had no significant impact on work or life; Effective: there was a certain deformity, slight claudication accompanied by pain, unable to perform physical labour; Ineffective: joint stiffness, defect or infection.

Total effective rate = (recovered + markedly effective + effective) / total number of cases × 100%.

## Results

Comparison of clinical efficacy between the two groups The total efficacy of the observation group is 91.67%, which is significantly higher than that of the control group (66.67%), and the difference has statistical significance (P < 0.05) (Table 1).

**Table 1:** Comparison of clinical efficacy between the two groups

Group	Number of cases	Reco vered	Markedly effective	Effective	Ineffective	Total effective rate (%)
Control group	24	9	5	2	8	66.67
Observation group	24	12	7	3	2	91.67

*P*						4.547
χ^2^						< 0.05

### Comparison of preoperative and postoperative AOFAS scores between control and observation groups

A total efficiency as high as 91.67% was showed in observation group, which is distinctly better than the effective rate of control group (66.67%, P<0.05); the postoperative AOFAS score of the two groups are higher than that before treatment, and the AOFAS score of control group is significantly lower than that of observation group (P < 0.05) (Table 2).

**Table 2:** Comparison of preoperative and postoperative AOFAS scores between the two groups (score, □ ± s)

Group	Number of cases	Before the operation	After the operation	t	*P*
Control group	24	42.08 ± 4.29	53.04 ± 6.79	6.685	< 0.05
Observation group	24	41.75 ± 5.31	66.28 ± 7.51	13.066	< 0.05

*t*		0.237	6.407		
*P*		0.814	< 0.05		

### Comparison of preoperative and postoperative fracture-healing time and hospital stays between the two groups

The hospitalized duration and healing time in observation group are obviously shorter than those in control group (P < 0.05) indicating potential less time and medical costs (Table 3).

**Table 3:** Comparison of preoperative and postoperative fracture-healing time and hospital stay between the two groups (□ ± s)

Group	Number of cases	Fracture-healing time (months)	Hospital stay (days)
Control group	24	4.18 ± 1.25	8.28 ± 2.54
Observation group	24	3.19 ± 1.04	3.57 ± 0.97

*P*		2.983	8.487
χ^2^		< 0.05	< 0.05

### Comparison of postoperative complications between two groups

Five cases of postoperative complications were recorded in control group (one case of talar necrosis, two cases of incision infection, two cases of traumatic arthritis; there are only two cases of complications in the observation group, all of which are traumatic arthritis. There is no statistical significance in the overall incidence rate of postoperative complications between control and observation groups were comparable and showed no significant difference (χ^2^ = 1.505, *P* > 0.05).

### Typical Case

**Figure 1:**
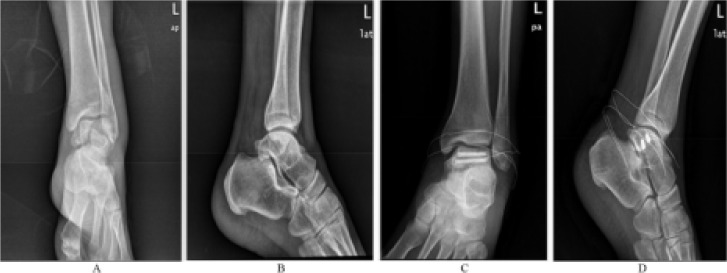
Comparison the results of preoperative and postoperative X-ray examination

A male patient (52-year-old) was admitted to hospital because of the “swelling, pain, and dysfunction of the left foot for 5 hrs caused by fall”. Physical examination: the left ankle was bruised and swollen with tenderness and faintly touchable bone friction. The activity of the left ankle joint was limited, the distal blood supply and toe movement of the left lower limb was good, and the sensation of shallow skin was normal. After admission, relevant examinations were supplemented. DR and CT of left foot both showed left talus fracture. After admission, the patient suffered from limb immobilization, thus detumescence with symptomatic and supportive treatment was delivered. Arthroscopic reduction and countersunk screw internal fixation of left talus fracture was also performed, and the operation process was successful. Infection prevention, functional exercise, detumescence, and symptomatic and supportive treatments were delivered postoperatively. Re-examination of the DR image showed good alignment of fracture end and stable internal fixation. The swelling of the left foot ankle gradually subsided, mobility was obviously improved after operation, the recovery went well, so the patient was discharged after clinical cure. The patient was followed up after discharge, and was satisfied with the clinical effect (Figure 1).

## Discussion

Because of the particularity of anatomical part and shape of the talus, the incidence of fracture in this part is not high in clinical practice, but with the increase of traffic accidents and work-related accidents, the incidence of fracture appears an upward trend. Open reduction and internal fixation (ORIF) has gradually become the preferred treatment for talar fractures, but this procedure is highly invasive, takes longer for the patient to recover, and may affect the patient's talar blood supply during surgery 8,9,10. Conservative treatment was not effective for talar cartilage injury, and traditional surgical treatment requires incision and local osteotomy to completely expose the talar roof, which was more traumatic^11^,^12^,^13^,^14^. With the development of minimally invasive techniques, arthroscopic techniques have been widely applied, such as internal fixation with countersunk nails after fracture reduction under arthroscopy, and fresh avulsion fractures can be fixed with wire or countersunk nails after fracture reduction under arthroscopy, and post-fracture fragment extraction, loose body removal, and intra-articular cartilage fragment cleaning^15^,^16^.

The result of present investigation showed that total efficiency of control group is 66.67%, which is obviously lower than that of observation group (91.67%, P<0.05). The hospitalized duration and fracture-healing time of control group were obviously longer than those in observation ones *(P*<0.05), consistent with the results in relevant study 12.

Small incision: in general, arthroscopic treatment can be completed with only two small incisions, which can effectively avoid the occurrence of complications such as incision infection caused by large-area skin incision. In this study, a total of 5 patients (20.83) had complications in the control group, and 2 patients (8.33) had complications in observation group. The patients involved in observation group did not have incision infection after operation.

The postoperative recovery time was short: in this study, the hospitalized duration and fracture-healing time in control group were obviously longer than those in observation ones *(P*<0.05). This may be ascribed to the smaller incision when arthroscopic technique was applied to treat patients, which can reduce the stripping of the tissues around the fracture site of the patient, improve the retention of original tissues, and have less damage to the nerves, blood vessels, muscles and other tissues around the fracture site, which is beneficial to the postoperative fracture healing and postoperative rehabilitation. The operation has less trauma and less pain for the patient, rehabilitation training can be performed in early postoperative period, which is helpful for the recovery of ankle joint function of the patient. In this study, AOFAS was significantly increased in the patients of both groups after the intervention, but the increase was significantly greater in observation group (P<0.05), thus confirms the above view. Although this procedure has many advantages mentioned above, the following problems should be paid attention to in clinical application^17^,^18^,^19^:

(1) The injury site should be thoroughly examined by imaging techniques before the operation to confirm the type of talus fracture and the degree of injury, combined with arthroscopic exploration to further confirm the diagnosis;

(2) The ankle joint space is small and the structure of the tibiotalar joint plane is concave-convex, so a certain traction force should be maintained during the operation to increase the joint space and facilitate the operation; and

(3) Early postoperative rehabilitation exercise training is also a key to accelerate the recovery of patients. Early postoperative appropriate exercise training is helpful to recover the blood supply at the injury site, which has a promoting effect on postoperative swelling and ankle function recovery.

## Conclusion

In conclusion, arthroscopic internal fixation with countersunk head nail for talus fracture can significantly improve the efficacy and ankle joint function, shorten the fracture-healing time and hospital stays, without increasing the incidence of complications, compared with the internal fixation and traditional open reduction.

## Data Availability

The datasets analysed during the current study are available from the corresponding author on reasonable request.
